# Retroperitoneal robot-assisted resection of a lower posterior mediastinal benign schwannoma using a transdiaphragmatic approach: A case report

**DOI:** 10.1097/MD.0000000000021765

**Published:** 2020-09-18

**Authors:** Jie Qin, Taile Jing, Ping Wang, Dan Xia, Shuo Wang

**Affiliations:** Department of Urology, First Affiliated Hospital, Zhejiang University School of Medicine, Hangzhou, Zhejiang Province, China.

**Keywords:** retroperitoneal, robotic, schwannoma, transdiaphragmatic

## Abstract

**Introduction:**

Neurogenic tumors are the most frequent neoplasms of the lower posterior mediastinum. Traditionally, lower posterior mediastinal tumors are excised by video-assisted thoracic surgery. However, the available robotic treatment for the lower posterior mediastinum tumors to date are rare. Herein, we report a case of a right lower posterior mediastinal tumors successfully treated with retroperitoneal robot-assisted surgery using a transdiaphragmatic approach.

**Patient concerns:**

A 54-year-old male patient without any symptoms was admitted into our department with a right lower posterior mediastinal paravertebral tumor that was detected during a medical check-up.

**Diagnosis:**

A right lower posterior mediastinal paravertebral tumor.

**Interventions:**

Retroperitoneal robot-assisted resection using a transdiaphragmatic approach was performed.

**Outcomes:**

The patient was treated with retroperitoneal robot-assisted surgery using a transdiaphragmatic approach and remained disease-free throughout a 6-month follow-up. His postoperative course was uneventful. Histopathological examination revealed a benign schwannoma.

**Conclusion:**

Our initial experience showed that retroperitoneal robot-assisted resection of a lower posterior mediastinal tumor using a transdiaphragmatic approach is technically feasible and can be considered a potential alternative for either video-assisted thoracic surgery or a thoracotomy.

## 1 Introduction

The most frequent neoplasms of the lower posterior mediastinum are neurogenic tumors. For the lower posterior mediastinal neurogenic tumors, video-assisted thoracic surgery (VATS) is recommended. Over the last 2 decades, the robotic system revolutionized laparoscopic surgery. These systems allowed for the substitution of laparoscopic complex procedures due to the enhanced visual information and improved ergonomics. The available robotic treatment for the lower posterior mediastinum are neurogenic tumors to date are rare.^[1–4]^ Herein, we report a case of a right lower posterior mediastinal paravertebral benign schwannoma successfully treated with retroperitoneal robot-assisted surgery using a transdiaphragmatic approach.

## 2 Case report

**Figure 1:**
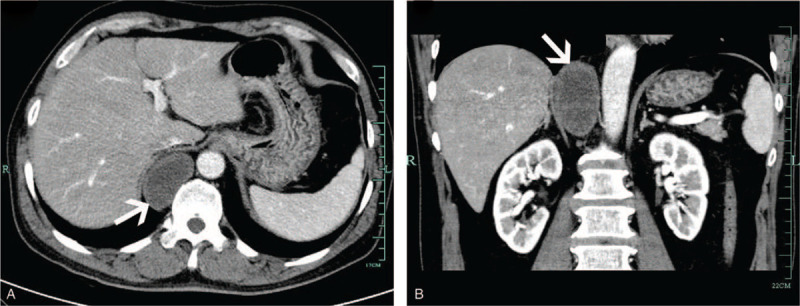
(A) Axial CT image showing the posterior mediastinal mass (white arrows); (B) Coronal CT image showing the posterior mediastinal mass (white arrows).

A 54-year-old man was admitted into our department with a right adrenal tumor that was detected during a medical check-up. Abdominal ultrasound was performed revealing a large adrenal tumor. The patient had normal vital signs and no underlying disease. The physical examination was unremarkable. Laboratory investigations including those for complete adrenal endocrinologic evaluation also showed no abnormalities. The computer tomography (CT) scan revealed a 7.0 × 6.0 × 5.0 cm neoplasm, which was a right lower posterior mediastinal paravertebral tumor and not a right adrenal tumor (Fig. 1). Additionally, the magnetic resonance imaging (MRI) showed no invasion of the tumor to any adjacent structures. Upon diagnosis of benign nature of the mass, a retroperitoneal robot-assisted resection was planned.

The patient was placed in a modified flank position under general anesthesia. The first incision was created over the iliac crest at the midaxillary line. After blunt dissection with the finger, a balloon dilatation apparatus was used to expand a working space in the retroperitoneum as previously described.^[5]^ Under guidance with the finger, we placed an 8-mm robotic trocar under the subcostal margin in the posterior axillary line. The second 8-mm robotic trocar was placed at the subcostal anterior axillary line, another 12-mm trocar was placed at the level of iliac crest in the anterior axillary line. We placed a 12-mm trocar above the iliac crest in the midaxillary line as a robotic camera port. After the pressure of the cavity was maintained at 10 to 14 mm Hg, a da Vinci (Intuitive Surgical, CA) robot was then docked.

**Figure 2:**
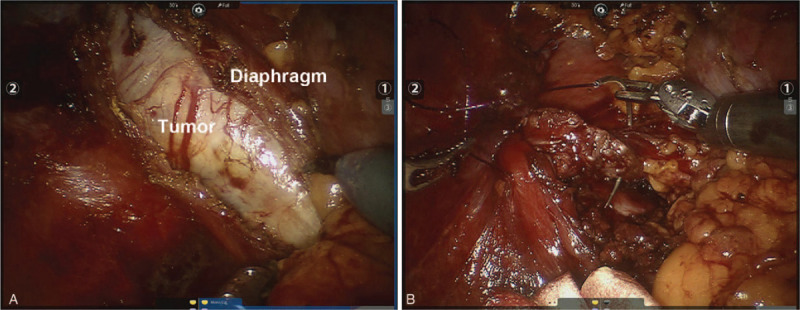
(A) Intra-operative view of the lower posterior mediastinal mass; (B) Intra-operative view of the suture of separated diaphragm after resection of the posterior mediastinal tumor.

**Figure 3:**
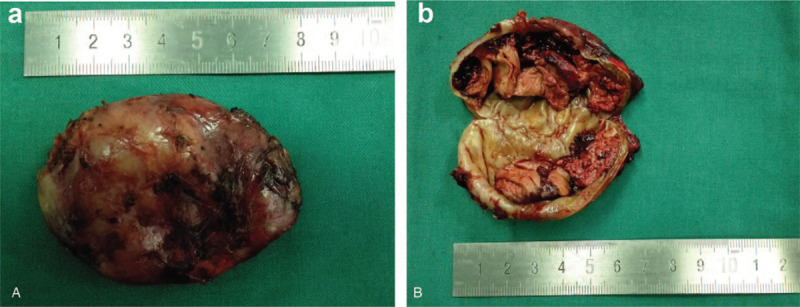
(A) A gross view of the removed Tumor; (B) The cut surface of the excised lower posterior mediastinal tumor.

Initially, the retroperitoneal fat was cleared and retrieved routinely. Then, the Gerotas fascia was opened longitudinally. After complete dissection of the non-vascular plane between the renal fat capsule and the psoas muscle, we located the mass, pushing the diaphragm upward. We incised the diaphragmatic muscle longitudinally and isolated the mass from the right pleura completely (Fig. 2). After 3 nerve roots were cut, the mass was removed. The diaphragmatic incision was then closed with 2–0 Prolene sutures. A retroperitoneal rubber drainage tube was placed at the suprarenal bed. No chest tube was placed. All tissue specimens were extracted in an endoscopic bag and retrieved through the 12 mm assistant port (Fig. 3). Histologic examination of frozen sections revealed schwannoma.

The total operation time was 135 minutes. The estimated blood loss was 50 ml. His postoperative course was uneventful and no postoperative complications were recorded. On the first postoperative day, the chest radiograph showed a well-expanded lung. The retroperitoneal drainage tube was removed after 72 hours. The patient was discharged on the third postoperative day, tolerating a regular diet. Follow-up at 3 month and 6 months after surgery, no focal sensory or motor deficits were found on neurological examination. The final pathological diagnosis of the resected specimen was a benign schwannoma.

## 3 Discussion

Schwannoma is a tumor derived from Schwann cells that surround peripheral nerve fibers. The etiology of schwannomas remain unclear.^[6–8]^ Schwannoma can affect any nerve trunk or any organ, but it occurs predominantly in the upper extremities. Less than 9% of schwannomas found in the mediastinum, but this tumor is the most common neurogenic tumor of the lower posterior mediastinum. Mediastinal schwannoma is usually found during routine medical check-ups and is further defined using computed tomography or magnetic resonance imaging. In adults, schwannomas are usually solitary and benign. Intraspinal extension occurs in about 10% of all posterior mediastinal schwannomas. To exclude intraspinal invasion, magnetic resonance imaging of the spine at the level of the mass is necessary. In this case, MRI showed no intraspinal invasion.

The treatment of choice for lower posterior mediastinal tumors is surgical resection. Traditionally, lower posterior mediastinal tumors are excised by standard posterolateral thoracotomy. One of the limiting factors associated with conventional thoracotomy is the morbidity and the cosmesis and pain of the large incision that impairs the respiratory muscles. The first thoracoscopic approach for resection of posterior mediastinal tumors was described by Landreneau et al in 1992.^[9]^ Compared to the conventional thoracotomy, video-assisted thoracic surgery has benefits include less postoperative pain, shorter postoperative hospital stays, and a better cosmetic appearance. These studies showed video-assisted thoracic surgery is the preferred treatment for posterior mediastinal benign neurogenic tumors.

There are some reported cases of lower posterior mediastinal tumor resection using a transdiaphragmatic approach by laparoscopic surgery in the literature.^[10–14]^ They were all used the transperitoneal approach. However, we have reported the first case of a lower posterior mediastinal benign schwannoma that was successfully retroperitoneoscopic resected using a transdiaphragmatic approach.^[15]^ Compared with video-assisted thoracic surgery, the laparoscopic approach is associated with a decreased anesthetic morbidity. By the laparoscopic surgery, we avoided placing a double-lumen endotracheal tube.

Laparoscopic resection of a lower posterior mediastinal tumor is always a challenge to the ergonomics of surgery. The main problem of laparoscopic complex surgical procedures is the limitations of the two-dimensional vision and range of motion of the laparoscopic instruments. The da Vinci Surgical System allows the urologists to perform complex surgical procedures, offering better visualization (the three-dimensional visualization), a tremor filtration and increased freedom of movement of the instruments. Robotic resection of posterior mediastinal tumors have been reported by neurosurgeons, urologists and cardio-thoracic surgeons.^[1–4]^

To our knowledge, there has been only one report in the literature on the surgical management of a lower posterior mediastinal tumor that was successfully retroperitoneoscopic resected using a transdiaphragmatic approach.^[16]^ Moskowitz et al reported a successful transdiaphragmatic robotic-assisted laparoscopic resection of a lower posterior mediastinal tumor via a retroperitoneoscopic laparoscopic approach. The urologist dissection of the spinal tumor retroperitoneoscopically. After the robot was docked, the neurosurgeon resected the tumor robotically. The total operative time was 5.3 hours, estimated blood loss was less than 50 ml, and the patient was discharged on postoperative day 2. They resected the tumor successfully with the combination of the pure retroperitoneoscopic approach and the robotic technology. Our case was done with a pure robotic technology. The total operative time was 135 minutes, estimated blood loss was 50 ml, and the patient was discharged on the third postoperative day.

Our initial experience showed that retroperitoneal robot-assisted resection of a lower posterior mediastinal tumor using a transdiaphragmatic approach is technically feasible with excellent perioperative outcomes. Because of the rarity of lower posterior mediastinal tumors, experience in the retroperitoneal robot-assisted laparoscopic surgery, is recommended. In conclusion, this novel technique represents a feasible and safe alternative to conventional laparoscopic approaches in the treatment of lower posterior mediastinal tumors. However, our study is limited by the nature of being a retrospective case report. Further long term follow-up in larger cohorts of patients are needed for further studies to determine the role of the retroperitoneal robot-assisted surgery using a transdiaphragmatic approach.

## Author contributions

**Conceptualization:** Jie Qin, Ping Wang, Dan Xia, Shuo Wang.

**Investigation:** Jie Qin, Taile Jing, Dan Xia, Shuo Wang.

**Resources:** Jie Qin, Taile Jing.

**Supervision:** Dan Xia, Shuo Wang.

**Writing – original draft:** Jie Qin, Taile Jing, Ping Wang.

**Writing – review & editing:** Jie Qin, Dan Xia, Shuo Wang.

## Abbreviations

Abbreviations: VATS = video-assisted thoracic surgery, CT = computed tomography, MRI = magnetic resonance imaging.
